# Different Responses of Growing Season Ecosystem CO_2_ Fluxes to Rain Addition in a Desert Ecosystem

**DOI:** 10.3390/plants12051158

**Published:** 2023-03-03

**Authors:** Xiaotian Xu, Bo Wu, Fang Bao, Ying Gao, Xinle Li, Yanli Cao, Qi Lu, Junliang Gao, Zhiming Xin, Minghu Liu

**Affiliations:** 1 Institute of Desertification Studies, Chinese Academy of Forestry, Beijing 100091, China; 2 Institute of Forestry and Pomology, Beijing Academy of Agriculture and Forestry Sciences, Beijing 100093, China; 3 Key Laboratory of Desert Ecosystem and Global Change, State Administration of Forestry and Grassland, Beijing 100091, China; 4 The Experimental Center of Desert Forestry of the Chinese Academy of Forestry, Bayannur 015200, China; 5 Dengkou Desert Ecosystem Research Station of Inner Mongolia, Bayannur 015200, China

**Keywords:** desert ecosystem, rain addition, CO_2_ fluxes, CO_2_ sink, nonlinear response

## Abstract

Desert ecosystem CO_2_ exchange may play an important role in global carbon cycling. However, it is still not clear how the CO_2_ fluxes of shrub-dominated desert ecosystems respond to precipitation changes. We performed a 10-year long-term rain addition experiment in a *Nitraria tangutorum* desert ecosystem in northwestern China. In the growing seasons of 2016 and 2017, with three rain addition treatments (natural precipitation +0%, +50%, and +100% of annual average precipitation), gross ecosystem photosynthesis (GEP), ecosystem respiration (ER), and net ecosystem CO_2_ exchange (NEE) were measured. The GEP responded nonlinearly and the ER linearly to rain addition. The NEE presented a nonlinear response along the rain addition gradient, with a saturation threshold by rain addition between +50% and +100%. The growing season mean NEE ranged from −2.25 to −5.38 μmol CO_2_ m^−2^ s^−1^, showing net CO_2_ uptake effect, with significant enhancement (more negative) under the rain addition treatments. Although natural rainfall fluctuated greatly in the growing seasons of 2016 and 2017, reaching 134.8% and 44.0% of the historical average, the NEE values remained stable. Our findings highlight that growing season CO_2_ sequestration in desert ecosystems will increase against the background of increasing precipitation levels. The different responses of GEP and ER of desert ecosystems under changing precipitation regimes should be considered in global change models.

## 1. Introduction

Arid regions dominated by desert ecosystems occupy 35% of the Earth’s surface and store 15% of the organic carbon of the world [1,2]. However, the role of desert ecosystems in the global carbon cycle has long been underestimated due to long-term drought stress, sparse vegetation, low soil nutrient levels, and low CO_2_ fluxes. Recently, temperate shrublands have been found to dominate the interannual variability of the global terrestrial carbon cycle [3], and desert ecosystems have been reported to play a considerable role as a CO_2_ sink [4,5,6]. However, due to great uncertainties and a lack of research, whether desert ecosystems are CO_2_ sources or sinks remains controversial [7]. Global circulation models predict that the total amount of precipitation in mid and high latitudes will show a rising trend in the near future, with an increasing frequency of extreme precipitation events [8,9]. Future climate scenarios predict changes in the precipitation regimes in the desert regions of northwestern China, following an increasing trend [6,8,9,10]. For example, based on the RCP8.5 scenario, increases in mean annual precipitation of up to 25%, 50% [11], and even more than 100% [12] are expected in certain desert regions at the middle and end of the 21st century compared with the end of the 20th century.

In arid regions, soil water is the main factor limiting the diversity and vigor of perennial plant species and shaping the structure, function, and diversity of ecosystems [13]. Changes in precipitation amounts and patterns (e.g., magnitude, interval, and seasonality) will affect soil water availability and hence influence the physiological and ecological processes of plants growing in xeric ecosystems [14,15,16,17]. Increased precipitation stimulates ecosystem CO_2_ uptake through photosynthesis and CO_2_ release via respiration, subsequently influencing the net ecosystem CO_2_ exchange (NEE), which reflects the overall CO_2_ sink function of ecosystems. Previous studies have shown that increased precipitation can enhance CO_2_ fluxes and productivity more significantly than decreased precipitation across various terrestrial ecosystems [18]. However, different vegetation types may respond differently to similar precipitation changes [19,20]. Although numerous studies have been conducted to evaluate the effects of precipitation variations on ecosystem-level CO_2_ fluxes through changing water availability [17], most of them (more than 75%) [21] primarily focused on forest and grassland ecosystems [22,23,24].

Accordingly, we still lack data sets on how water controls carbon cycling, especially regarding the magnitudes of CO_2_ exchange fluxes in desert shrublands, and the results of existing studies remain largely various. For example, a study in the Chihuahuan Desert found that increased precipitation enhanced soil CO_2_ release and vegetation CO_2_ uptake, consequently enhancing NEE (becoming more negative in convention for the sign) [25]. Results from a desert grassland also showed that a large precipitation pulse enhanced NEE of both low- and high-cover areas [26]. However, in a study by Snyder et al., summertime irrigation shifted the soil water potential but did not increase either CO_2_ uptake or plant growth in shrubs in the Great Basin and the Mojave Desert [27]. The results from a rangeland experiment in Arizona indicated that NEE during precipitation pulses was strongly controlled by CO_2_ efflux and driven by species effects and soil microclimate [28,29]. In the Sonoran Desert, it has been found that the precipitation pulse effects on soil respiration were enlarged in fine-textured vegetated region, especially when the antecedent conditions were dry [30]. The results from an experiment in a temperate desert indicate that whether increased precipitation enhanced or dampened NEE depends on the responses of precipitation timing and ephemeral plant growing season coupling [31]. Given that many factors, including plant community type, soil type, seasonality of precipitation, and climate, may play a huge role, the explanation of the inconsistent conclusions of the above-mentioned studies were limited. As the review by Beier et al. mentioned, an important way to better clarify the responding mechanisms of desert CO_2_ exchange under changing precipitation regimes is to conduct multilevel treatments considering the current and future climate scenarios [21]. From the findings of the previous studies, it has been well accepted generally that ANPP and CO_2_ uptake increased with the increase in water availability, leveling off when extreme wet conditions are reached [32]. Both field experiments and model predictions found the response of CO_2_ fluxes rates showed similar concave down curves [33,34,35,36,37], with a saturation pattern. However, multiple treatment precipitation amounts involving comparisons between increases and decreases were only considered in a few studies [38,39], which is also the case for the responses of soil respiration to precipitation with gradients in grassland [14,40,41] and desert ecosystems [42]. In this sense, focusing on the integrates of CO_2_ fluxes along precipitation gradients, which are involved in extreme precipitation conditions that refer to future climate scenarios, will be helpful for clarifying the patterns of CO_2_ fluxes responding to precipitation change.

In this study, we conducted a manipulated experiment along a rain addition gradient to evaluate the CO_2_ fluxes responses of a *Nitraria tangutorum*-dominated desert ecosystem in Inner Mongolia, northwest China, investigating the responses of the ecosystem CO_2_ exchange processes. We attempted to answer the following questions: How do ecosystem CO_2_ fluxes (i.e., GEP, ER, and NEE) respond along a rain addition gradient? Does additional rain increase or decrease the net ecosystem CO_2_ uptake in a desert ecosystem? Given that desert ecosystems are often characterized with vegetation constrain and resource limitation, we tested the following hypotheses: (a) With increasing annual growing season rainfall, GEP, ER, and NEE will all increase nonlinearly, with different saturation thresholds. (b) The photosynthesis process (GEP) will benefit more than the respiration process (ER), leading to an enhanced net ecosystem CO_2_ exchange (NEE).

## 2. Results

### 2.1. Rain Addition Treatments and SWC Changes

**Figure 1:**
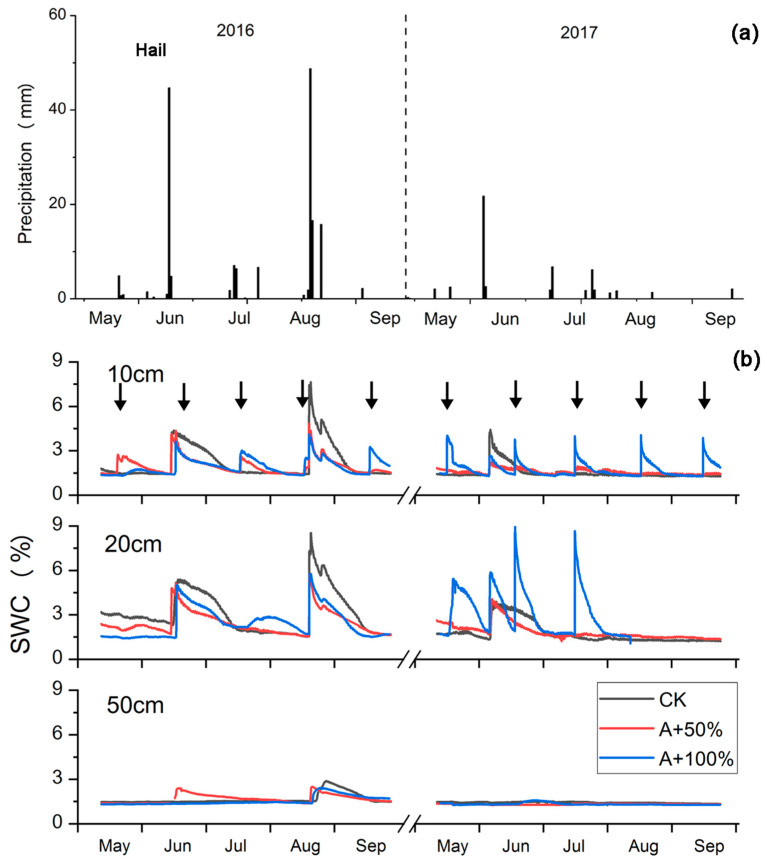
Daily precipitation (**a**) and soil water content (SWC) at depths of 10, 20, and 50 cm (**b**) in the two growing seasons in 2016 and 2017 on the *N. tangutorum*-dominated nabkhas. Black arrows represent rain addition treatments. C = control, A + 50% = rain addition by 50%, and A + 100% = rain addition by 100%. SWC at depth of 20 cm in August and September of 2017 was missing due to instrument failure.

The natural rainfall in the growing seasons (May to September) in 2016 and 2017 was 167.2 and 54.5 mm, respectively. Compared to the historical mean growing season value of 124 mm from 1961 to 2006 (Figure S1), they represented a wet year (2016) and a dry year (2017). On June 13, 2016, a hailstorm occurred in the study area, with a rainfall of 44.8 mm, causing a large number of leaves in the nabkhas to fall off (more than 90%; Figure 1a). The growing season rainfall was increased by the rain addition from 167.2 and 54.5 mm in 2016 and 2017 to 238.9 mm and 127 mm for A + 50% treatment, respectively, and for 311.4 and 199.5 mm for the A + 100% treatment.

As shown in Figure 1b, the SWC of the nabkhas in the growing season was low. If there was no large rainfall event, the SWC was mostly around 1.5%. Rain addition resulted in a rapid increase in SWC, reaching a peak within a short time, followed by a gradual decrease. In the case of heavy natural rainfall, the effect of rain addition and natural rainfall may be superposed. In addition, both A + 50% and A + 100% treatments resulted in an increase in SWC at a depth of 10 cm by 40.8% and 66.7% compared to the control plots, respectively. While at the depth of 20 cm, only A + 100% resulted in an increase in SWC. At the depth of 50 cm, the two treatments had little effect on SWC in the two growing seasons; this was also the case for natural rainfall events larger than 40 mm.

### 2.2. Response of Ecosystem CO_2_ Fluxes to Rain Addition

**Figure 2:**
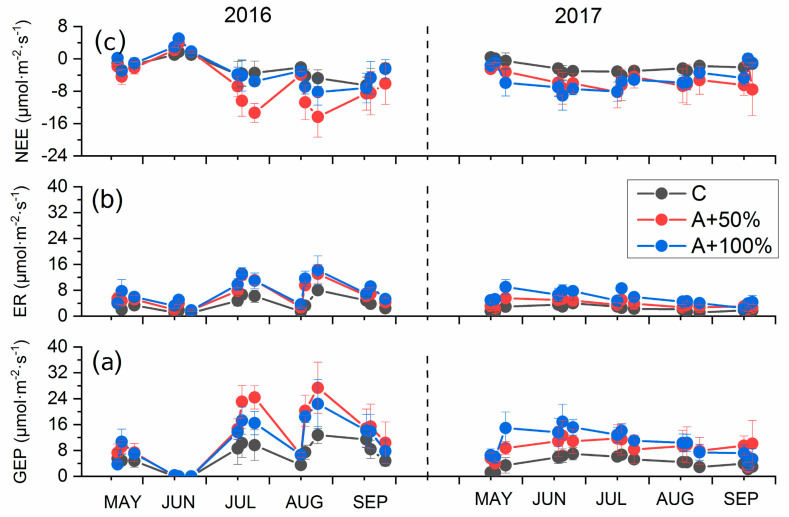
Changes in the CO_2_ fluxes ((**a**), gross ecosystem photosynthesis, GEP; (**b**), ecosystem respiration, ER; and (**c**), net ecosystem CO_2_ exchange, NEE) on the *N. tangutorum*-dominated nabkhas in two growing seasons under rain addition treatments. Error bars represent standard errors. C = control, A + 50% = rain addition by 50%, and A + 100% = rain addition by 100%. The monthly, interannual, and overall effects can be found to Table S1, Table 1 and Table 2, respectively.

During the 9-day-a-month measurement period (from 1 day before to 7 days after the rain addition treatment), significant pulse responses of CO_2_ fluxes to rain addition were observed (Figure 2 and Figure S4). In most cases, GEP increased more persistently and steadily after the treatment, while ER increased rapidly after rain addition and then decreased to a level close to that prior to rain addition, showing a response peak (Figure S4), and NEE showed a similar pattern with GEP. In addition, natural rainfall events around the 9-day windows have certain influences on the response pulses of CO_2_ fluxes. As shown in Figure S4, in May and August in 2016 and May in 2017, there were natural rainfalls >2 mm within the measurement periods, where CO_2_ fluxes 7 days after the rain additions were enhanced. In June, July, and September in 2016, there were natural rainfalls >2 mm before rain additions, which may have enhanced the CO_2_ fluxes 1 day before the rain additions. Among them, only the two heaviest rainfall events in June and August in 2016 may have induced sound impacts (Figure 1 and Figure S4).

The pulse responses of CO_2_ fluxes showed seasonal variation, with different response amplitudes in different months (*p* < 0.05, Table S1). Correspondingly, all CO_2_ fluxes also changed greatly in different months (*p* < 0.001, Table 1). In 2016, the magnitude of all components of CO_2_ fluxes in July and August were about 60% higher (more negative for NEE) than those in other months, and the lowest CO_2_ fluxes were observed in May except June. In June 2016, the GEP was close to zero due to the loss of most of the leaves, caused by hail. In 2017, the peaks of the magnitude of CO_2_ fluxes were advanced to June and July, due to a 22 mm rainfall event in June. In September of these two growing seasons, when the leaves of *N. tangutorum* began to wither and fall, the magnitude of all CO_2_ fluxes rapidly decreased (Figure 2).

Overall, rain addition significantly affected all components of the ecosystem’s CO_2_ flux (*p* < 0.001, Table 1). The mean values of GEP, ER, and NEE across the two growing seasons for the control were 5.20, 2.95, and −2.25 μmol CO_2_ m^−2^ s^−1^, respectively. For the A + 50% treatment, they were 10.48, 5.01, and −5.38 μmol CO_2_ m^−2^ s^−1^, and for the A + 100% treatment, they were 10.27, 6.57, and −3.70 μmol CO_2_ m^−2^ s^−1^. Compared with the control, the magnitude of GEP, ER, and NEE values for the A + 50% treatment increased by 102%, 73% and 139%, respectively (more negative for NEE), and those for the A + 100% treatment increased by 98%, 123% and 64%, respectively (Figure 2). Across all treatments, the mean growing season values of GEP and ER significant decreased from 9.95 and 5.89 μmol CO_2_ m^−2^ s^−1^ in 2016 to 7.85 and 3.85 μmol CO_2_ m^−2^ s^−1^ in 2017, respectively, showing significant interannual variation (*p* < 0.05) (Figure 2). In contrast, NEE values in the growing season in 2016 (−3.55 μmol CO_2_ m^−2^ s^−1^) and 2017 (−4.00 μmol CO_2_ m^−2^ s^−1^) were not significantly different, without interannual variation (Table 2).

### 2.3. Response of CO_2_ Fluxes to Total Rainfall Amount (TRA)

**Figure 3:**
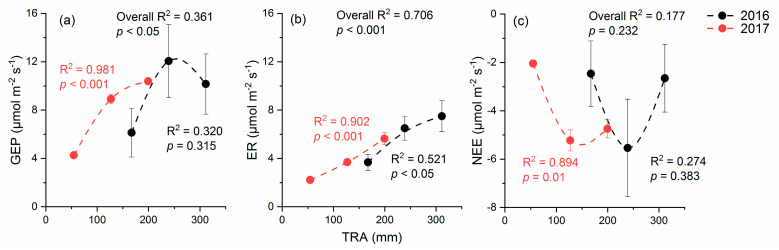
Regression relationships between total rainfall amounts (TRAs) and mean growing season CO_2_ fluxes ((**a**), gross ecosystem photosynthesis, GEP; (**b**), ecosystem respiration, ER; and (**c**), net ecosystem CO_2_ exchange, NEE) in 2016 and 2017. For GEP and NEE, nonlinear regressions are shown, while for ER, linear regressions are shown according to smaller *p* values during regression. The regression equations with statistical significance were GEP in 2017 (y = −1.339 + 0.119x − 3.03 × 10^−4^x^2^), ER in 2016 (y = 0.027x − 0.448) and 2017 (y = 0.024x + 0.853), and NEE in 2017 (y = 2.769 − 0.107x + 3.94 × 10^−4^x^2^).

In 2016 and 2017, the mean CO_2_ fluxes during the growing season were fitted with the total rainfall amount (TRA, the sum of added rainfall and natural rainfall) as the x-axis (Figure 3). For GEP (Figure 3a), the promotion effect of the A + 50% treatment was stronger than that of the A + 100% treatment and exhibited a quadratic nonlinear response pattern, while the relationship was significant only in 2017 (*p* < 0.01). For ER (Figure 3b), A + 100% had a stronger promotion effect than A + 50% and showed a significant linear response pattern (*p* < 0.05 in 2016 and *p* < 0.001 in 2017). The fitting with NEE was similar to GEP, exhibiting a nonlinear but not statistically significant pattern (*p* > 0.05) (Figure 3c).

### 2.4. Influences of Environmental Factors on CO_2_ Fluxes

**Figure 4:**
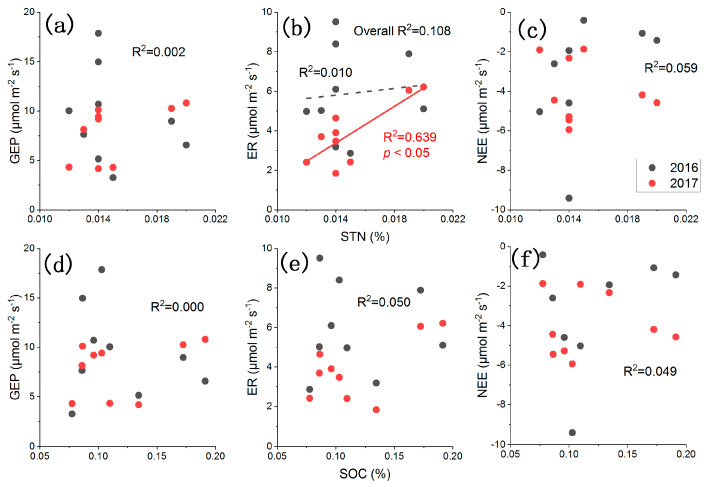
Relationships between the CO_2_ fluxes ((**a**,**d**), gross ecosystem photosynthesis, GEP; (**b**,**e**), ecosystem respiration, ER; and (**c**,**f**), net ecosystem CO_2_ exchange, NEE) and soil nutrient factors (STN = soil total nitrogen, SOC = soil organic carbon).

Based on the results of the regression analysis, among the environmental factors which may affect CO_2_ fluxes, the air temperature in the chamber and soil moisture at a depth of 20 cm had better relationship with the CO_2_ fluxes than the soil temperature and soil moisture at 10 cm depth. Air temperature was positively correlated with ER and GEP and negatively correlated with NEE (*p* < 0.05). The relationship between ER and soil temperature at a depth of 10 cm was poor (*p* > 0.05), while the relationships of GEP and NEE with soil temperature at 10 cm were better (*p* < 0.05, except for GEP in 2016). Soil moisture at 10 cm had a weak influence on CO_2_ fluxes and was only significantly correlated with ER in 2017 (*p* < 0.001) and NEE in 2016 (*p* < 0.05), while soil moisture at 20 cm had stronger influence on CO_2_ fluxes (*p* < 0.05 except for GEP in 2016 and NEE in 2017) (Table 3). Further multiple regression analyses indicated that these changes in CO_2_ fluxes were primarily driven by the changes in soil moisture at 20 cm, especially in 2017. Soil moisture at 10 cm only exhibited stronger effects than 20 cm in 2017, while temperatures were generally ineffective except for ER. The effects of soil nutrient factors (total nitrogen, STN, and organic carbon, SOC) on CO_2_ fluxes were also evaluated, with generally no significant relationships (*p* > 0.05) except for STN and ER in 2017 (*p* < 0.05) (Figure 4). Further partial relationship analysis showed that, after controlling the meteorological drivers (air temperature, soil temperature, SWC), the general nonsignificant pattern did not change, with the largest partial correlation coefficients of 0.551 (*p* = 0.06) being between STN and ER.

## 3. Discussion

### 3.1. Differential Response Patterns of CO_2_ Fluxes

In our study, all components of the CO_2_ flux increased in magnitude with rain addition, but their response patterns were different. In line with our hypothesis, the enhancement of GEP showed a nonlinear response, and its possible saturation threshold was between A + 50% and A + 100%. However, ER showed different response patterns with GEP, with a linear response, which did not support our hypothesis. As a results, NEE showed a nonlinear response (Figure 3).

As a shrub of the genus *Nitraria* in the family Tribulus (Zygophyllaceae), *N. tangutorum* is endemic to China and mainly distributed in Inner Mongolia, Gansu, Shaanxi, Xinjiang, and other provinces in northwestern China; it also grows in arid Gobi deserts and at the edges of sandy deserts [43]. Because of its strong tolerance and adaptability to drought, salinity, cold, and wind, it has become the main dominant species of desert vegetation and a good sand-fixing plant in the arid region of northwestern China [44,45,46]. In the *N. tangutorum* distribution area, long-term annual average precipitation is about 80–300 mm, which means that the total precipitation corresponding to the A + 100% treatment in this study reached the upper limit of precipitation in the natural distribution area of *N. tangutorum*. *N. tangutorum* may adapt to low amounts of water available, with the photosynthesis capacity parameters saturated at a high precipitation level [47]. Therefore, it is not surprising that GEP and NEE tended to show saturation thresholds at a TRA value close to 300 mm (Figure 3). Compared to ER, the patterns of GEP and NEE against the total rainfall amounts were substantially scattered, and although the plants received more precipitation in 2016 than 2017, the GEP almost unchanged in each plot (Figure 3). This indicated that the extra water did not play a significant role. Besides the possible limited photosynthesis capacity, another key factor regulating the interannual variations of CO_2_ fluxes was the difference of meteorological conditions. The year of 2016 was much wetter than 2017, with two heavy natural rainfall events >40 mm, largely promoting the CO_2_ fluxes. Nevertheless, one of these two rainfall events was a hailstorm, causing great damage to plant leaves, which limited the overall seasonal GEP. The extra water from these heavy rainfall events may not be fully utilized by the plants, reducing the water use efficiency, and they weakened the relationship between GEP and environmental factors (Table 3). In addition, these two rainfall events were both around the 9-day windows of the CO_2_ flux measurement, leading to uncertainties of our results. The conclusion of our study may still need more validation in the future works.

The mechanism underlying the differences in photosynthesis (GEP) and respiration (ER) response patterns can be explained by their different response modes to rainfall. Generally, as an assimilation process, the response of photosynthesis to rainfall lags behind that of respiration because it requires water to infiltrate into the soil depth available to the plant and to last long enough to stimulate the plant to absorb water [48,49]. In our study region, the root system of *N. tangutorum* can distribute into deep soil of about 3 m below the aboveground shoots, with about 1–2 m in the sandy nabkhas and 1 m in the clay plain below the sand dunes [50]. However, respiration responds rapidly after rainfall, because the soil microbial community in the top layer will respond rapidly to even small rainfall events [48,51]. Therefore, large rainfall can directly translate to an increase in respiration, while the increase in photosynthesis can be steadier and more colimited by other factors. Previous studies showed that in semiarid grasslands, the nonlinear response of respiration is mainly due to substrate limitation [22,36,41], and increased soil water availability may translate water limitation into nutrient limitation [52,53,54]. However, our data in this study and our previous publications [55,56] suggested that on the *N. tangutorum* nabkhas with poor sandy soil, rain addition may not bring substrate and nutrient limitation. First, SOC and STN generally increased among the rain addition gradient (*p* < 0.05, Table 4), showing a strong positive relationship between SWC and STN (R^2^ = 0.577, *p* < 0.05). Second, rain addition promoted the growth of *N. tangutorum* [55] as well as of annual plants (*Salsola collina* and *Agriophyllum squarrosum*) in this field, which can be indicated by the increase in total vegetation cover (Table 4). Third, our previous study in this field had also demonstrated that rain addition increased microbial biomass carbon [56], indicating that the promotion of vegetation growth on the nabkhas may enhance microbially mediated litter decomposition, thus providing more nutrients for the soil. In addition, a former study in an *N. tangutorum* desert ecosystem confirmed that soil respiration increased linearly along a rain addition gradient [42]. Therefore, for ER, the linear response can be attributed to sufficient amounts of substrate and the rapid promotion of ER by the instantaneous reaction of soil microbes. These results indicate that under the TRA condition in this study, respiration did not reach the saturation threshold.

### 3.2. Rain Addition Enhanced NEE of the Desert Ecosystem

From the perspective of the growing season NEE of the *N. tangutorum* nabkhas when plants are leafed out and growing, the mean values ranged from −2.25 to −5.38 μmol CO_2_ m^−2^ s^−1^, showing net CO_2_ uptake effect. After rain addition, CO_2_ fluxes were enhanced, and NEE were promoted by up to more than 200% in magnitude (Figure 2). Under completely different natural rainfall conditions, based on the observation of the three CO_2_ flux components for 2 years, the growing season NEE was stable and robust.

The enhancement effect of NEE was the result of the response of GEP and ER to rain addition. In our study, the magnitude of all CO_2_ flux components were promoted by rain addition, and the response amplitude of GEP was greater (from 5.20 to 10.48 μmol CO_2_ m^−2^ s^−1^) than that of ER (2.95 from 6.57 μmol CO_2_ m^−2^ s^−1^). This is in agreement with studies of the *N. tangutorum* desert ecosystem in Minqin, Gansu Province, and with other studies conducted on the same rain addition experimental platform used in this study [42,47,55,57]. In these studies, leaf photosynthesis and soil respiration rates were enhanced, and plant shoot growth also increased significantly with rain addition. Our results indicate that NEE was more closely related to GEP (R^2^ = 0.76) than ER (R^2^ = 0.25), similar to previous studies in other regions [34]. Previous studies suggested that plant photosynthesis is predominately limited by available water, nutrients, and leaf photosynthesis capacity [36,58], with greater sensitivity than respiration, which is often determined by belowground activities of roots and microbes colimited by soil substrate and nutrient [59,60]. Therefore, under changing water availability, GEP can show greater variability and had greater impact on NEE. In arid ecosystems, such as grasslands and deserts, similar findings have been widely reported, where increased precipitation stimulated higher CO_2_ uptake than release [26,61,62,63]. These results highlight that an increase in water supply would favor CO_2_ sequestration by promoting aboveground plant activity (see also [58]), which could back up modeling studies reporting that more extreme rainfall events may benefit arid ecosystems by providing more available water [16].

Although natural precipitation varied greatly in 2016 and 2017, in both growing seasons, rain addition generally enhanced the NEE in magnitude. In June 2016, an unexpected hailstorm resulted in a large loss of leaves and, consequently, a sharp decline in photosynthesis. However, from the perspective of the entire growing season, NEE was not significantly affected. This is mainly because the hailstorm brought a large amount of rain, but also because the negative impact of hail on plants [64] and the positive impact of the sufficient water supply in the early growing season on the net CO_2_ uptake of the ecosystem [65,66] cancel each other out. The year 2017 was a dry year, but limited natural precipitation and low SWC did not significantly reduce the growing season NEE, even in the control plots without rain addition (Figure 1, Table 1). Generally, arid regions show a high interannual variability of precipitation. Based on the historical records from 1961–2006, the average variability of growing season precipitation in our study site was 31.87%, ranging from −69.68 to +112.82% (37.6 to 263.9 mm) (Figure S1). Therefore, the vegetation community in this region may show intrinsic insensitiveness to short-term precipitation fluctuations and other environmental variations.

### 3.3. Implications for the Carbon Cycling of Desert Ecosystems

Our findings based on the long-term rain addition experiment showed that the *N. tangutorum* desert ecosystem acts as net CO_2_ uptake during the growing season, with a significant increase under the A + 50% and A + 100% treatments. Although natural precipitation in 2016 and 2017 varied greatly, rainfall in the growing seasons of 2016 and 2017 was 134.8 and 44.0%, respectively, of the historical mean value of 124 mm (1961–2006). Although the hailstorm in June 2016 caused the loss of more than 90% of the leaves of *N. tangutorum*, the NEE remained stable. Therefore, against the background of increasing precipitation levels, CO_2_ sequestration in desert ecosystems will increase. However, our study primarily focused on the fluxes during the 5-month growing season, while the actual annual NEE can be codetermined by how rain addition treatments influence the respiration (both for the plant and soil) during the 7-month nongrowing season when most CO_2_ fluxes are toward the atmosphere, which means the whole picture of annual ecosystem CO_2_ exchange still need more investigations.

The NEE presented a nonlinear response along the rain addition gradient, and its possible saturation threshold was between A + 50% and A + 100%. Previous studies have indicated that both the GEP and ER of grassland ecosystems showed a nonlinear increase with increasing rainfall levels [22,36,41]. In contrast to the grassland ecosystem, GEP showed a nonlinear and ER a linear response along the rain addition gradient in the desert ecosystem (Figure 3). In this sense, the different response patterns of photosynthesis and respiration of desert ecosystems to changing precipitation regimes should be considered in a global change model.

## 4. Materials and Methods

### 4.1. Study Area

The experiment was carried out in a desert ecosystem on the northeast edge of the Ulan Buh Desert, Dengkou County, Inner Mongolia Autonomous Region, China (E106°43′, N40°24′, 1050 m a. s. l., Figure S2). The area is characterized by an arid continental climate, with a mean annual temperature of 7.8 °C and a mean annual precipitation of 145 mm, concentrated from July to September. Mean annual potential evaporation is 2327 mm, and the frost-free period is 136–205 days. The vegetation type is temperate desert shrubland with a vegetation cover of 20–35%. The community develops on nabkhas (oval sand dunes with deep-rooted shrubs) and is solely dominated by *N. tangutorum*. Each nabkhas was 0.8–1.8 m in height, with a short and long axis length of 5–9 m. The root system of *N. tangutorum* can distribute about 3 m below the aboveground shoots, with about 1–2 m in the sand dunes and 1 m in the clay plain below the sand dunes [50]. Generally, nabkhas scatter on a red clay ground to form a mosaic desert landscape and are 3–8 m away from each other. Occasionally, some species, e.g., *Salsola collina* and *Agriophyllum squarrosum*, also occur in the community, albeit at lower numbers. The soils covering the nabkhas consist of >90% sand and are thus categorized as sandy soils according to the China Soil Taxonomy System [67].

### 4.2. Experimental Design

According to historical records obtained from a local weather station (downloaded from the National Meteorological Information Center, http://data.cma.cn/ (accessed on 22 November, 2018)), the annual amount of precipitation has increased markedly from 1961–2006, which was more evident in the growing season (May to September, Figure S1). This trend was consistent with the future trend predicted by models for this region [8,68,69]. The rain addition experiment began in 2008 and is still ongoing based on a mean annual precipitation of 145 mm (1961–2006) at the study site. We used a completely randomized design with three treatments of rain addition by 0% (C), 50% (A + 50%), and 100% (A + 100%), and three replicates for each treatment (nine plots in total). In each plot of a circle area with a diameter of 12 m (113.04 m^2^), only one natural nabkha is located in the center of the plot. The nabkhas in all plots were similar regarding growing condition and size. The morphological characteristics of the nabkhas (length, height, area, and volume) as well as the plant cover are shown in Table 4. The distance between every two plots was more than 5 m to avoid possible interactions. During the growing season (May to September), rain addition was applied on the 15th of each month, and the rain amount added was 14.5 mm per month for A + 50% (72.5 mm per year) and 29 mm per month for A + 100% (145 mm per year). Water was pumped into a tank from a well near the plots and then irrigated into the plots via an irrigation system with a water-pump, water meters, and spraying arms (Figure S3a). The irrigation systems were installed on the top of the nabkha in the center of the plot. Two spraying arms can rotate freely about 0.3 m above the shrub crown on the top of nabkha to evenly spray the simulated rainwater over the treatment area. To reduce water evaporation at the time of rain addition, water addition was conducted in the early morning when the air temperature was relatively low and the air above the land surface was usually calm. The groundwater in the experimental site was below a depth of 5 m and did therefore not affect plant growth.

### 4.3. Ecosystem CO_2_ Flux Measurements

In each plot, the middle position of the southern slope of the nabkhas was selected. The vegetation at this position grows well and distributes evenly and can represent the general vegetation coverage on the nabkhas well, and it was used to measure the CO_2_ fluxes. In April 2016, a square stainless-steel frame (0.50 m × 0.50 m) was inserted into the soil at the selected measurement site on all nabkhas to a depth of 3 cm (Figure S3b). During installation, soil disturbance was largely avoided. In the growing seasons of 2016 and 2017, we used an infrared gas analyzer (IRGA, Li-8100, LI-COR Inc., Lincoln, NE, USA) attached to a transparent measuring chamber (0.50 m × 0.50 m × 0.90 m) to measure NEE and ecosystem respiration (ER) (Figure S3c). The frames provided a flat base between the soil surface and the chamber, and the box covered the entire vegetation within the frame. Two plastic tubes with a length of about 1 m connected the analyzer and the box to make sure that the air that was pulled through the tubing to the IRGA was recirculated back to the chamber during each measurement. During the measurements, two small electric fans installed within the box worked continuously to ensure that the CO_2_ was evenly mixed and the air temperature in the box did not increase too quickly. Each measurement of 2 min included 20 s of prepurge, a dead band of 30 s to achieve steady state, 10 s of postpurge, and a ventilation interval of 30 s before the next measurement. Therefore, the effective measurement time was a period of 1 min, with 60 consecutive recordings of CO_2_ concentrations at 1 s intervals under a relatively steady condition (change of air and soil temperature less than 0.2 °C, which were monitored immediately during the measurement using the JM 222 hand thermometer (Jinming, Tianjin, China) and the auxiliary temperature sensor of Li-8100, respectively). After measuring NEE, the chamber was lifted and vented to recover the CO_2_ concentration for 30 s, placed on the base frame, and covered with a cardboard box, creating a dark environment for the chamber by blocking the sunlight to measure ER. Since light (and hence photosynthesis) was eliminated, the value of the second CO_2_ exchange measurement represented ER.

In each month, we measured NEE and ER between 10:00 a.m. and 12:00 a.m. 1 day before, 1 day after, and 7 days after rain addition. Every three treatment plots were taken as one set, and the three sets of plots were measured in a consistent order.

After measuring NEE and ER, the gross ecosystem photosynthesis (GEP) was calculated as follows:

GEP = ER − NEE(1)

Based on this equation, the values of both GEP and ER were positive, while for NEE, positive values represent CO_2_ release, and negative values represent CO_2_ absorption.

### 4.4. Soil Property Measurements

We used an EM-50 (decagon, Pullman, WA, USA) to measure the soil water content (SWC) of three typical plots (representing three treatments) at the depths of 10, 20, and 50 cm. Due to probe failure, data were lost for the depth of 20 cm of A + 100% after 10 August 2017. Since the EM-50 ECH_2_O EC-5 sensor is less accurate when used on sandy soils with low SWC, it was corrected based on previous studies under similar soil conditions [70].

For all nabkhas in all plots, soil nitrogen and organic carbon contents were determined, as shown in Table 4, using soil samples collected at depths of 0–20 cm. Five uniformly distributed replicates per plot were obtained using a soil core sampler (5 cm in diameter) after the end of rain addition in September 2016. All soil samples were air-dried and ground to pass a 100-mesh (mesh size of 150 μm) screen for analysis. Soil nitrogen content was determined by the Auto-Kjeldahl method (Kjektec System 1026 Distilling Unit, Hoganas, Sweden), while organic carbon was determined using the sulfuric acid and an aqueous potassium dichromate (K_2_Cr_2_O_7_) mixture with external heating. Measurements were conducted at the Research Center of Plant Ecology, Institute of Botany, Chinese Academy of Sciences, Beijing, China.

### 4.5. Statistic Analysis

Repeated measures analyses of variances (ANOVAs) were conducted to examine the interannual variability in the seasonal means of the ecosystem CO_2_ fluxes (NEE, ER, and GEP) when combined with the rain addition treatments. Repeated measures ANOVAs were used again to examine the treatment effects over the growing season each year. The between-subject effect was rain addition, and the within-subject effect was the month of the year and its interactions with rain addition. We also used repeated measures ANOVAs to investigate the effects of rain addition in each measurement month; the within-subject effects were the measurement dates and the interactions with the rain addition. Linear and exponential regressions, multiple regression, and partial correlation analysis were conducted between the CO_2_ fluxes and the environmental factors (air temperature, soil temperature, soil moisture at a depth of 10 cm and 20 cm, STN, and SOC). All analyses were performed using the statistical software SPSS 21.0 (IBM, Amonk, NY, USA).

## 5. Conclusions

In summary, our field experiment demonstrated that CO_2_ fluxes of the *N. tangutorum* desert ecosystem can be enhanced by the increased rainfall amounts, with the GEP responding nonlinearly and the ER linearly to rain additions. Correspondingly, the NEE presented a nonlinear response along the rain addition gradient, with enhanced CO_2_ uptake of −2.25 to −5.38 μmol CO_2_ m^−2^ s^−1^. These findings indicate that, in the forthcoming climate change scenarios, the growing season CO_2_ sequestration in desert ecosystems will be more active when the precipitation increases, but this trend may reach a saturation when the precipitation doubles due to the different response patterns of photosynthesis and respiration. We suggest that these influencing factors should be considered in the future global change models to improve the accuracy of prediction.

## Figures and Tables

**Table 1:** Results (F values) of repeated measures ANOVA on the effects of the measurement months, rain addition treatments, and their interactions on the CO_2_ fluxes (a, gross ecosystem photosynthesis, GEP; b, ecosystem respiration, ER; and c, net ecosystem CO_2_ exchange, NEE) during the growing seasons of 2016 and 2017.

	CO_2_ Flux	2016	2017
Month (M)	GEP	35.974 ***	13.709 ***
	ER	24.564 ***	17.487 ***
	NEE	31.505 ***	10.864 ***
Treatment (T)	GEP	4.319 *	6.219 **
	ER	8.756 **	15.993 ***
	NEE	3.678 *	3.106
Interaction (M × T)	GEP	1.861	1.391
	ER	1.000	1.160
	NEE	2.118	1.169

Notes: *, **, and *** represent significant differences at *p* < 0.05, *p* < 0.01, and *p* < 0.001, respectively. Bold values indicate a significant difference at *p* = 0.05. Sample sizes (*n*) of each variable were 9 and 15 for month and treatment, respectively.

**Table 2:** Results (F values) of repeated measures ANOVA on the effects of the measurement years, rain addition treatments, and their interactions on the seasonal means of the CO_2_ fluxes (a, gross ecosystem photosynthesis, GEP; b, ecosystem respiration, ER; and c, net ecosystem CO_2_ exchange, NEE).

	Variable	F
Year (Y)	GEP	5.236 *
	ER	55.496 ***
	NEE	0.798
Treatment (T)	GEP	24.496 ***
	ER	58.812 ***
	NEE	12.773 ***
Interaction (Y × T)	GEP	1.975
	ER	2.157
	NEE	2.633 ***

Notes: * and *** represent significant differences at *p* < 0.05 and *p* < 0.001, respectively. Bold values indicate a significant difference at *p* = 0.05. Sample sizes (*n*) of each variable were 9 and 6 for year and treatment, respectively.

**Table 3:** Relationships between the CO_2_ fluxes (a, gross ecosystem photosynthesis, GEP; b, ecosystem respiration, ER; and c, net ecosystem CO_2_ exchange, NEE) and environmental factors.

Year	Factor	GEP		ER		NEE	
		R^2^	*p* Value	R^2^	*p* Value	R^2^	*p* Value
2016	Air temperature	0.051	**0.017**	0.178	**<0.001**	0.035	**0.03**
	Soil temperature	0.027	0.083	0.005	0.429	0.055	**0.006**
	Soil moisture at 10 cm	0.026	0.090	0.002	0.576	0.043	**0.016**
	Soil moisture at 20 cm	0.000	0.848	0.033	**0.040**	0.129	**<0.001**
2017	Air temperature	0.100	**0.001**	0.077	**0.004**	0.047	**0.024**
	Soil temperature	0.055	**0.007**	0.004	0.505	0.033	**0.038**
	Soil moisture at 10 cm	0.004	0.486	0.103	**<0.001**	0.009	0.275
	Soil moisture at 20 cm	0.143	**<0.001**	0.383	**<0.001**	0.031	0.059

Note: The regressions between ER, GEP, and temperature are exponential, and the others are linear. Bold values indicate a significant difference at *p* = 0.05. Sample sizes (*n*) of each variable were 135 for each year.

**Table 4:** Characteristics of the experimental field in the *N. tangutorum* desert ecosystem.

	C	A + 50%	A + 100%
East–west length of the nabkhas (m)	5.75 ± 0.76	6.30 ± 0.89	7.00 ± 1.34
South–north length of the nabkhas (m)	6.45 ± 1.03	6.05 ± 0.69	8.83 ± 1.83
Height of the nabkhas (m)	1.25 ± 0.18	1.18 ± 0.07	1.40 ± 0.23
Area of the nabkhas (m^2^)	30.51 ± 7.67	29.94 ± 5.06	51.64 ± 15.38
Volume of the nabkhas (m^3^)	13.98 ± 4.95	11.94 ± 2.45	26.11 ± 10.20
Plant cover (%)	25.00 ± 3.54 a	26.25 ± 5.54 a	33.75 ± 4.73 a
Relative cover of *N. t.*	0.74 ± 0.09 a	0.83 ± 0.06 a	0.63 ± 0.07 a
Canopy height (cm)	51.88 ± 3.73 a	60.69 ± 3.69 a	61.81 ± 2.95 a
Soil organic carbon (%)	0.104 ± 0.012 a	0.099 ± 0.006 a	0.157 ± 0.024 b
Soil total nitrogen (‰)	0.143 ± 0.009 a	0.140 ± 0.004 a	0.178 ± 0.013 b

Notes: C = control, A + 50% = rain addition by 50%, and A + 100% = rain addition by 100%. *N. t.* means *Nitraria tangutorum*. Data represent mean values ± standard errors; different letters within rows represent significant differences in the Duncan test. These data were measured in 2016.

## Data Availability

The data that support the findings of this study are available on request from the corresponding author.

## Supplementary Materials

The following supporting information can be downloaded at: https://www.mdpi.com/article/10.3390/plants12051158/s1, Table S1: Results (F values) of repeated measures ANOVA on the effects of the rain addition treatments, measurement days relative to the treatment day and their interactions on NEE (net ecosystem carbon exchange), ER (ecosystem respiration), and GEP (gross ecosystem photosynthesis) during each month in 2016 and 2017; Figure S1: Geographical location of the study area; Figure S2: Variation trend of annual precipitation and precipitation in growing season before the experiment (1960–2006); Figure S3: Photo of (a) the simulated rain addition equipment on the top of a nabkha, (b) measurement position, and (c) assimilation box and Li-8100 analyzer; Figure S4: ER (ecosystem respiration) and GEP (gross ecosystem photosynthesis) on 1 day before, 1 day after, and 7 days after the rain addition treatment in the *N. tangutorum* desert ecosystem in the two growing seasons.
