# Supplementary material for: Different Responses of Growing Season Ecosystem CO2 Fluxes to Rain Addition in a Desert Ecosystem
Source: Plants (Basel). 2023 Mar 3;12(5):1158. doi: 10.3390/plants12051158 (PMC10005604; doi:10.3390/plants12051158)

Different Responses of Growing Season Ecosystem CO_2 Fluxes to Rain Addition in a Desert Ecosystem

Xiaotian Xu [1,5], Bo Wu [1,2,*], Fang Bao [1,2], Ying Gao [1,2], Xinle Li [3,4], Yanli Cao [1,2], Qi Lu [1,2], Junliang Gao [3,4], Zhiming Xin [3,4] and Minghu Liu [3,4]

[1] Institute of Desertification Studies, Chinese Academy of Forestry, Beijing 100091, China; arthurpku@163.com (X.X.); baofang@caf.ac.cn (F.B.); yinggao@caf.ac.cn (Y.G.); caoyanli@caf.ac.cn (Y.C.); luqi@caf.ac.cn (Q.L.)
[2] Key Laboratory of Desert Ecosystem and Global Change, State Administration of Forestry and Grassland, Beijing 100091, China
[3] The Experimental Center of Desert Forestry of the Chinese Academy of Forestry, Dengkou 015200, China; nxylxl@126.com (X.L.); gaojunliang1985@163.com (J.G.); xzmlkn@163.com (Z.X.); slzxlmh@sina.com (M.L.)
[4] Dengkou Desert Ecosystem Research Station of Inner Mongolia, Dengkou 015200, China
[5] Institute of Forestry and Pomology, Beijing Academy of Agriculture and Forestry Sciences, Beijing 100093, China
* Correspondence: wubo@caf.ac.cn; Tel.: +86-010-62824016

Tables

Table S1. Results (F values) of repeated measures ANOVA on the effects of the rain addition treatments, measurement days relative to the treatment day and their interactions on NEE (net ecosystem carbon exchange), ER (ecosystem respiration) and GEP (gross ecosystem photosynthesis) during each month in 2016 and 2017.

	Variable	2016					2017				
		May	Jun	Jul	Aug	Sep	May	Jun	Jul	Aug	Sep
Date	GEP	3.602	4.830	**7.903***	**16.626****	**24.246*****	**14.740***	2.302	**5.880***	**15.710****	2.183
	ER	0.031	**45.287*****	**10.436***	**18.108****	**13.825****	**22.051****	0.643	**5.574***	**5.135***	0.337
	NEE	**10.742***	**60.568*****	2.611	**8.826***	**16.477****	**7.641***	2.218	3.998	**7.923***	3.129
Treatment	GEP	0.512	3.245	2.105	2.706	0.341	3.309	1.506	0.593	0.934	0.887
	ER	1.314	**6.495***	3.596	**5.274***	2.048	**6.492***	2.593	**6.131***	3.176	1.816
	NEE	0.680	**7.661***	1.779	1.779	0.247	1.059	1.081	0.636	0.529	0.882
Date × Treatment	GEP	2.402	3.245	2.254	0.963	0.885	2.355	0.692	1.476	0.828	0.604
	ER	1.661	**5.738***	0.877	0.790	1.872	1.678	1.250	2.741	2.870	0.814
	NEE	0.350	**7.068***	1.695	1.191	0.764	2.674	0.150	1.297	0.900	1.200

Notes: *, **, and *** represent significant differences at $p < 0.05$, $p < 0.01$, and $p < 0.001$, respectively. Bold values indicate a significant difference at the $p = 0.05$ level. Sample sizes (n) of each variable were 9.

Figure captions

Fig. S1. Geographical location of the study area. Data source: https://viewer.esa-worldcover.org/worldcover

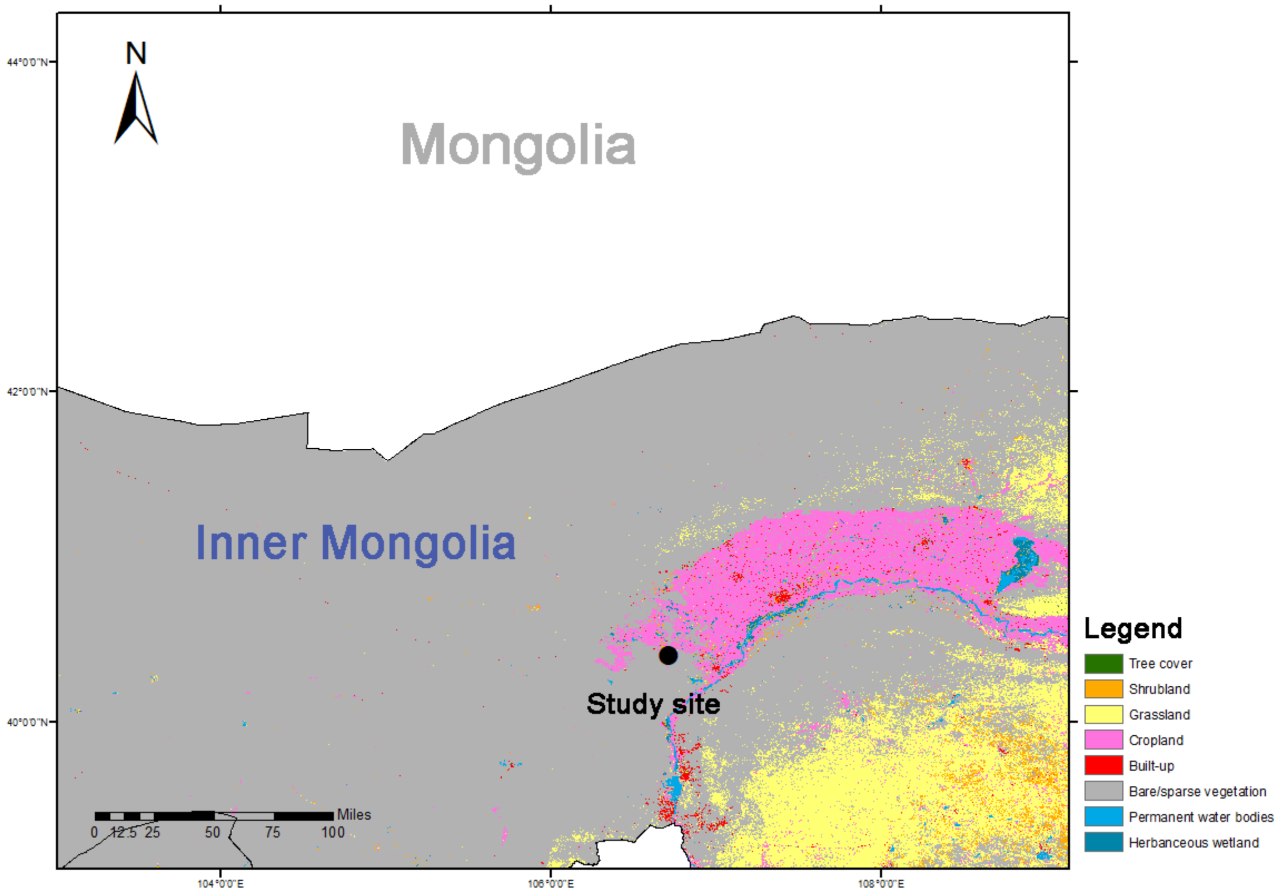

Fig. S2. Variation trend of annual precipitation and precipitation in growing season before the experiment (1960-2006). The data were obtained from the local meteorological station (E107° 00′, N40° 20′) near the experiment site (about 20 km away from the site).

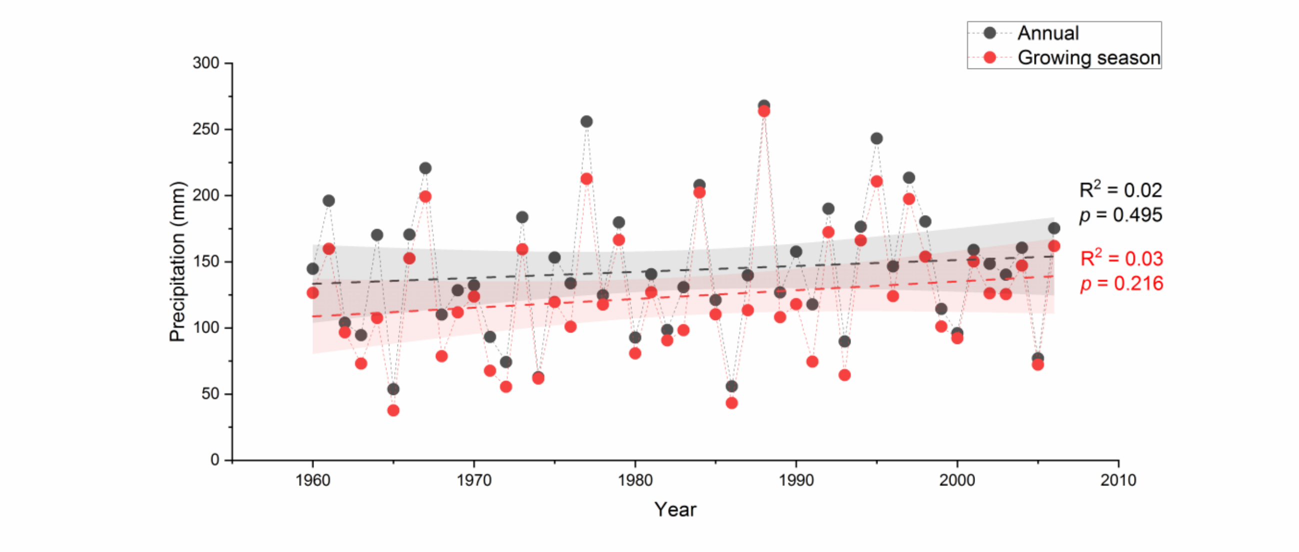

Fig. S3 Photo of (a) the simulated rain addition equipment on the top of a nabkha, (b) measurement position and (c) assimilation box and Li-8100 analyzer.

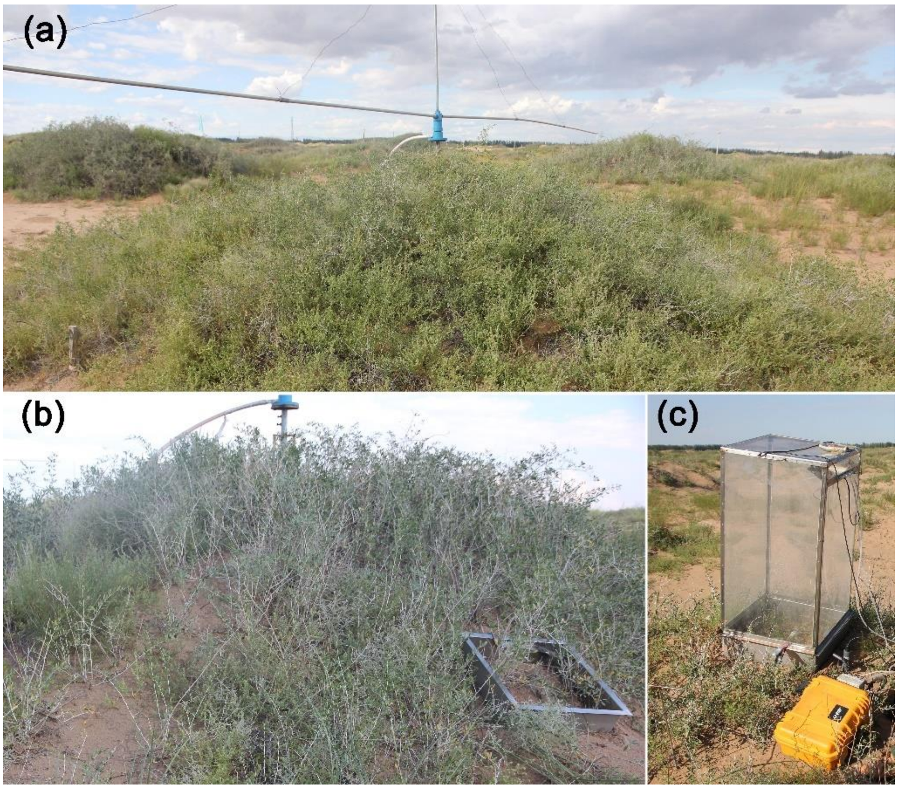

Fig. S4. ER (ecosystem respiration) and GEP (gross ecosystem photosynthesis) on the 1 day before, the 1 day and 7 days after the rain addition treatment in the *N. tangutorum* desert ecosystem in the two growing seasons. Dash lines represented the rain addition date, and numbers in brackets indicated the dates when natural rainfall events observed around the 9-day measurement periods.

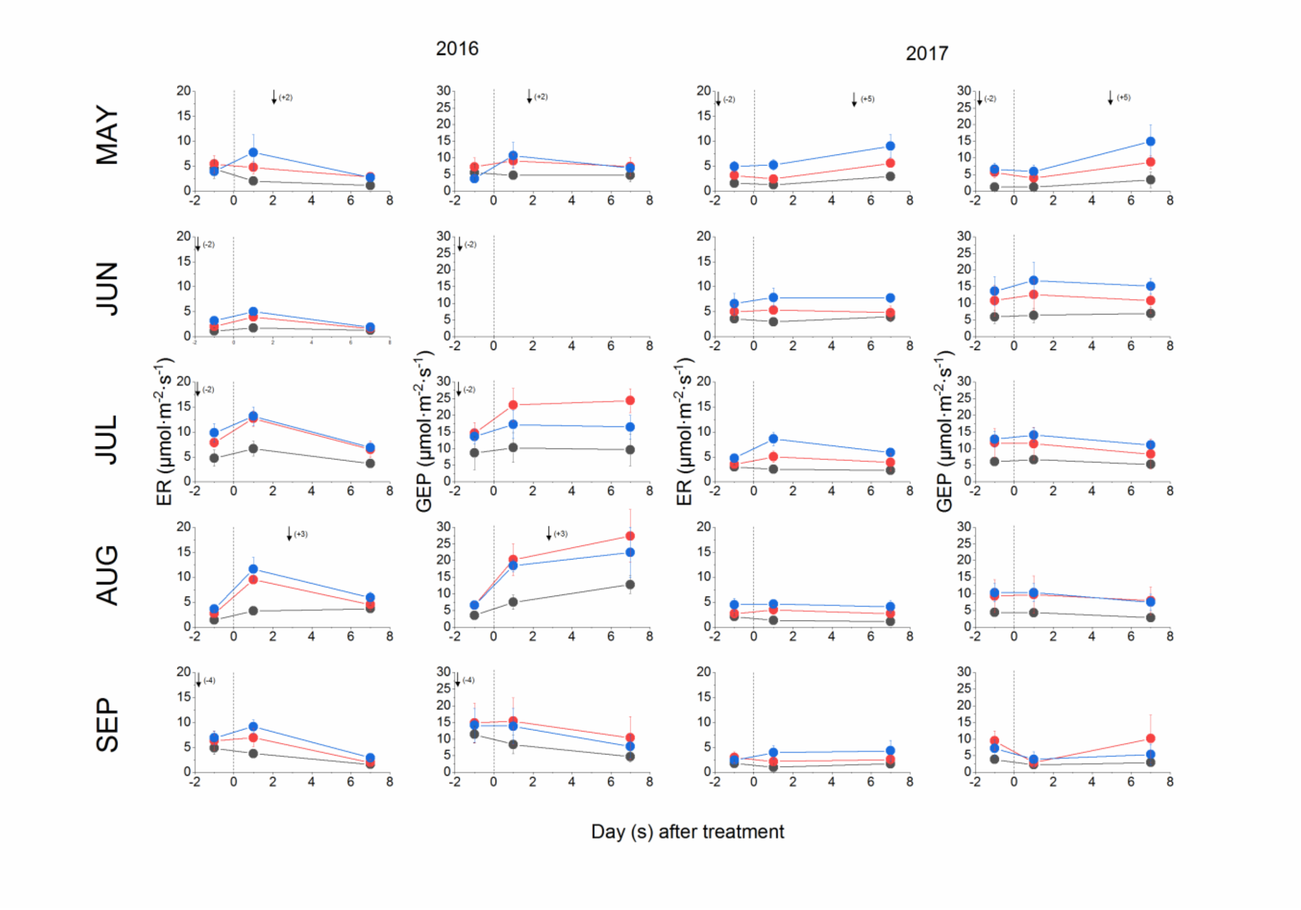

Supplement: Supplementary file 1 [file plants-12-01158-s001.zip › plants-2195708-supplementary.pdf]
